# Simulated Mars Gravity Impairs Intestinal Epithelial Barrier Integrity via Selective Modulation of Tight Junction Components

**DOI:** 10.3390/biom16050739

**Published:** 2026-05-18

**Authors:** Laura Benvenuti, Chiara Bertini, Gemma Marcelli, Chiara Ippolito, Valentina Citi, Roberto Giovannoni, Paola Iacopetti, Gaetana Gambino, Leonardo Rossi, Debora Angeloni, Diego Manzoni, Alessandra Salvetti

**Affiliations:** 1 Department of Translational Research and New Technologies in Medicine and Surgery, University of Pisa, 56126 Pisa, Italy; laura.benvenuti@phd.unipi.it (L.B.); diego.manzoni@unipi.it (D.M.); 2 Department of Physics, University of Trento, 38123 Trento, Italy; chiara.bertini@unitn.it; 3 Department of Clinical and Experimental Medicine, University of Pisa, 56126 Pisa, Italy; gemma.marcelli@med.unipi.it (G.M.); chiara.ippolito@unipi.it (C.I.); paola.iacopetti@unipi.it (P.I.); alessandra.salvetti@unipi.it (A.S.); 4 Department of Pharmacy, University of Pisa, 56125 Pisa, Italy; valentina.citi@unipi.it; 5 Department of Biology, University of Pisa, 56126 Pisa, Italy; roberto.giovannoni@unipi.it; 6 Interuniversity Center for the Promotion of the 3Rs Principles in Teaching and Research, 56122 Pisa, Italy; 7 Institute of Biorobotics, Scuola Superiore Sant’Anna, 56124 Pisa, Italy; debora.angeloni@santannapisa.it

**Keywords:** claudins, tight junctions, Mars, microgravity, STAT3, RPM, intestinal epithelial barrier

## Abstract

Future long-duration human space missions will expose astronauts to chronically reduced gravitational loading, a condition associated with oxidative stress and epithelial barrier dysfunction. The intestinal epithelial barrier depends on tight junctions (TJs), yet the impact of partial gravity on TJ composition, assembly, and claudin organization remains poorly defined. Here, we show that differentiated intestinal epithelial monolayers exposed to simulated Mars gravity undergo TJ ultrastructural remodeling, characterized by loss of apical membrane “kissing points” and widening of the paracellular space, accompanied by impaired barrier function. Simulated Mars gravity also induces oxidative stress and accumulation of cytoplasmic and nuclear lipid droplets, consistent with altered membrane and lipid homeostasis. At the molecular level, simulated Mars gravity promotes selective TJ changes, with significant downregulation—but not mislocalization—of barrier-forming claudins CLDN1 and CLDN3 and the scaffolding protein ZO-1, while CLDN2, CLDN4, CLDN7, CLDN12, CLDN23, and OCLN remain unchanged. STAT3 activation, but not ERK or NF-κB signaling, may be associated with these alterations and is consistent with a stress-adaptive remodeling response to oxidative stress under simulated Mars gravity. Overall, these findings identify simulated Mars gravity as a disruptor of intestinal barrier homeostasis and highlight TJ remodeling as a target for countermeasures to preserve gut integrity during deep-space missions.

## 1. Introduction

In recent years, growing interest has focused on future human space exploration missions to the Moon and, ultimately, to Mars and beyond; however, our understanding of the health risks associated with long-duration spaceflight remains incomplete. In particular, long-duration missions beyond low Earth orbit will expose astronauts to a combination of extreme environmental stressors, which pose substantial challenges to human physiological homeostasis [1,2,3].

In particular, altered gravitational loading and exposure to ionizing radiation are widely recognized as dominant contributors to spaceflight-related dysfunction, in part through increased generation of reactive oxygen species (ROS) [4]. Indeed, experimental evidence from both real and simulated microgravity demonstrates that spaceflight conditions perturb mitochondrial function and oxidative balance [5]. Mitochondrial impairment, disruption of mechanotransduction pathways, and weakening of antioxidant defenses act synergistically to promote ROS accumulation in microgravity, thereby perturbing cellular signaling and homeostatic regulation [6]. In particular, altered mechanotransduction has been shown to modulate ROS production through cytoskeletal remodeling, mechano-sensitive signaling pathways, and activation of redox enzymes [7]. Oxidative stress has been associated with a broad spectrum of spaceflight-induced alterations, including musculoskeletal degeneration, immune dysregulation, cardiovascular remodeling, and neurological dysfunction [6].

Data derived from spaceflight missions and ground-based simulated microgravity models also reveal marked alterations in intestinal physiology, including reduced mucin production, increased epithelial permeability, and shifts in gut microbiota composition [8,9]. Imbalanced ROS levels can favor microbial dysbiosis and increase the passage of microbial-derived products into the circulation, thereby promoting chronic low-grade inflammation and perpetuating oxidative stress-associated pathology [10].

At the cellular level, the integrity of the intestinal barrier relies on specialized intercellular junctional complexes that regulate epithelial cohesion, among which tight junctions (TJs) are of primary importance. Positioned at the apical region of epithelial cells, TJs control paracellular transport and restrict the diffusion of luminal antigens, toxins, and pro-inflammatory mediators [11]. Compromise of the TJ function is a common pathological feature of several gastrointestinal disorders, including inflammatory bowel disease, and is strongly associated with increased epithelial permeability and mucosal inflammation [12].

TJ components include occludin, junctional adhesion molecules (JAMs), zonula occludens (ZO) proteins, and claudins, which are key regulators of paracellular permeability [13]. Claudins constitute a multigene family comprising more than twenty-five members, each conferring distinct permeability properties to epithelial barriers in a tissue-specific manner [14]. Aberrant expression or mislocalization of individual claudins has been documented in intestinal inflammatory diseases, highlighting their essential role in preserving gut epithelial homeostasis [15,16]. In addition to their established barrier functions, claudins participate in a range of non-canonical cellular processes, including epithelial differentiation, intracellular signaling cascades, and cytoskeletal organization [17].

Despite the central role of TJ in maintaining intestinal epithelial barrier integrity, only limited data are available regarding TJ component regulation under microgravity conditions. Experimental studies have demonstrated that simulated microgravity induces a delay in the apical junctional localization of occludin and ZO-1, leading to impaired epithelial barrier function and increased paracellular permeability in intestinal epithelial cells [18]. Consistently, long-term exposure to simulated microgravity using a tail-suspension rat model resulted in significant intestinal barrier disruption, enlarged intercellular spaces, and downregulation of adhesion molecules such as claudin-1 protein, accompanied by increased intestinal permeability [19,20]. Similarly, a reduction in the expression of TJ proteins such as occludin, claudin-1, claudin-5, and E-cadherin has been demonstrated in the ileum of rats after hindlimb unloading for 21 days, and this reduction was closely associated with the activation of TLR4/MyD88/NF-κB signaling [8].

Collectively, these findings indicate that TJ protein expression is sensitive to altered gravity and that microgravity represents a critical but still underexplored regulator of epithelial barrier integrity. Given the functional diversity of the claudin family and their established involvement in intestinal inflammation and barrier dysfunction, a systematic investigation of claudin regulation and TJ remodeling in response to microgravity-induced oxidative and mechanical stress is warranted. As future human exploration of Mars represents a key strategic objective of current space programs, such studies are essential to elucidate the molecular mechanisms underlying spaceflight-associated intestinal barrier impairment and to identify targets for countermeasures aimed at preserving gut homeostasis during long-duration missions.

## 2. Materials and Methods

### 2.1. Cell Culture and Microgravity Experiments

Human colon carcinoma cells (Caco-2) were obtained from ATCC (HBT-37, ATCC, Manassas, VA, USA). Cells were cultured and maintained in Eagle’s minimum essential medium (EMEM) supplemented with 20% fetal bovine serum (FBS), 1% non-essential amino acids (100×), 1% L-glutamine, and 1% penicillin–streptomycin, in a humidified atmosphere of 5% CO_2_ at 37 °C. Cell culture medium and supplements were purchased from Sial (yourSial, S.I.A.L. Srl, Rome, Italy).

To generate a polarized monolayer, cells were seeded at a density of 6 × 10^4^ cells/cm^2^ onto 24-well Transwell inserts (Greiner Bio-One GmbH, Frickenhausen, Germany), previously coated with a thin layer of type I collagen (C8919, Sigma Aldrich, St. Louis, MO, USA) following the supplier’s instructions. After 24 h, Transwell inserts were transferred into 30 mL syringes filled with complete medium (three Transwell per syringe) to prevent shear stress caused by air bubbles. The syringes were then placed at the center of the Random Positioning Machine (RPM) (Yuri, Meckenbeuren, Germany) frame, which was programmed via the RPM Controller software Airbus DS (Rpm 2.0, software version 2.5.0.4) to simulate Mars gravity (0.38 g), to minimize gravitational variations. The RPM was stored in a cell incubator for the entire duration of the experiments, and the medium in the syringes was changed 2–3 times per week. Parallel control experiments were performed under normal Earth gravity (1 g).

### 2.2. Calcium Switch Assay

Caco-2 cells (15 × 10^4^) were seeded on 24-well Transwell inserts and cultured until confluence. To evaluate TJ assembly, cells were incubated for 24 h in MEM calcium-free (#11380-037 Gibco, Thermo Fisher Scientific, Waltham, MA, USA) supplemented with 1% non-essential amino acids (100×), 1% L-glutamine, and 1% penicillin–streptomycin, in the absence of FBS. Control cells underwent the same treatment in complete EMEM without FBS. Following this incubation, Caco-2 cells were either fixed immediately (time 0) or exposed to complete EMEM and stopped at various timepoints. At each time point, cells were washed with ice-cold 1× phosphate-buffered saline (PBS, yourSial, S.I.A.L. Srl, Rome, Italy) and fixed in 4% paraformaldehyde (PFA; Sigma Aldrich, St. Louis, MO, USA) for subsequent immunofluorescence analysis.

### 2.3. Transepithelial Electrical Resistance (TEER)

TEER was measured in control and simulated Mars gravity-cultured cells to assess epithelial barrier integrity. TEER was determined using an EVOM2 epithelial voltohmmeter (World Precision Instruments, Sarasota, FL, USA), which allows a non-invasive evaluation of Caco-2 monolayer integrity. Resistance values were expressed as Ω·cm^2^ and represent the integrity and tightness of intercellular junctions. Higher TEER values indicate enhanced tight junction organization and improved barrier function.

### 2.4. Cytofluorimetry (ROS and Lipid Content)

#### 2.4.1. ROS Assay

General oxidative activity was assessed using the 2′,7′-dichlorodihydrofluorescein diacetate (DCFH-DA, #D6883, Sigma-Aldrich, Merck, Darmstadt, Germany) probe. Caco-2 cells were cultured on Transwell inserts for 21 days to allow complete differentiation and then incubated with 20 µM DCFH-DA for 1 h. Cells were subsequently detached using Trypsin-EDTA (yourSial, S.I.A.L. Srl, Rome, Italy) and resuspended in 300 µL 1× PBS for cytometry analysis. Flow cytometry was performed on an Accuri C6 Plus (BD Biosciences, Franklin Lakes, NJ, USA). Event acquisition was performed as previously described by Gambino et al. [21].

#### 2.4.2. Lipid Content

Intracellular lipid content was assessed by Nile Red (Sigma-Aldrich, Merck, Darmstadt, Germany) staining. Caco-2 cells were cultured on Transwell inserts for 21 days to allow differentiation. A stock solution was prepared by dissolving Nile Red powder in DMSO to a final concentration of 2 mM. Cells were detached using Trypsin-EDTA, resuspended in 300 µL PBS containing 500 nM Nile Red, and incubated for 10 min at 37 °C. Stained cells were analyzed using an Accuri C6 Plus flow cytometer (BD Biosciences, Franklin Lakes, NJ, USA) as described by Gambino and colleagues [21].

### 2.5. Transmission Electron Microscopy

For each experimental condition, half of a Transwell insert containing differentiated Caco-2 cells was washed with 1× PBS and then processed as described in Gambino and coauthors [21]. Briefly, cells were fixed in 2.5% glutaraldehyde in 0.1 M cacodylate buffer (pH 7.2) for 2 h at 4 °C, post-fixed in 1% osmium tetroxide for 1 h at room temperature, then rinsed twice for 5 min each in cacodylate buffer and dehydrated at room temperature through a graded ethanol series. All reagents were purchased from Sigma Aldrich (St. Louis, MO, USA).

After dehydration, samples were embedded in epoxy resin (Sigma Aldrich, St. Louis, MO, USA) and finally polymerized at 60 °C for 72 h. Ultra-thin sections were obtained with a diamond knife on an Ultracut ultramicrotome (Reichert-Jung, Vienna, Austria) collected on Formvar-carbon-coated copper grids (Ted Pella, Inc., Redding, CA, USA), and stained with uranyl acetate and lead citrate (Ted Pella, Inc., Redding, CA, USA). Sections were observed with a Jeol 100 SX transmission electron microscope (Tokyo, Japan) (CIME, University of Pisa).

### 2.6. RNA Extraction

Total RNA was extracted from 21-day-differentiated Caco-2 cells using TRIzol™ LS Reagent (Invitrogen, Thermo Fisher Scientific, Waltham, MA, USA), according to the manufacturer’s instructions. To remove genomic DNA contamination, RNA samples were treated with DNase I (Invitrogen, Thermo Fisher Scientific, Waltham, MA, USA) for 30 min at 37 °C. RNA concentration and purity were assessed using a NanoDrop spectrophotometer (Thermo Fisher Scientific, Waltham, MA, USA).

### 2.7. Real-Time PCR

Quantitative real-time PCR was performed using the GoTaq^®^ qPCR Master Mix (#A6002; Promega, Madison, WI, USA) on aQuantStudio^TM^ 5 Real-Time PCR System (Thermo Fisher Scientific, Waltham, MA, USA). The thermal cycling conditions were initial denaturation at 95 °C for 2 min, followed by 40 cycles of 95 °C for 10 s, and 64 °C for 30 s. A melting curve analysis was carried out from 65 °C to 95 °C to confirm product specificity. All reactions were carried out in duplicate. Expression levels of target genes were normalized to the housekeeping *GAPDH* and *HPRT* genes. Relative gene expression was calculated using the 2^−ΔΔCt^ method. Data acquisition and analysis were performed withDesign & Analysis Software 2.8.0 (Thermo Fisher Scientific, Waltham, MA, USA). Primer sequences are reported in Table S1.

### 2.8. Western Blot Assay

Cells were lysed as follows: after 21 days of differentiation, Transwell inserts were washed with 1× PBS, and cells were detached with trypsin-EDTA. The obtained pellet was resuspended in 80 μL RIPA buffer (ab150634, Abcam, Cambridge, UK), supplemented with cOmplete™, Mini protease inhibitor cocktail (11836153001, Sigma-Aldrich, Merck, Darmstadt, Germany), and mechanically homogenized by pestling (100 strokes). The homogenate was then vortexed every 5 min for 30 min while maintained on ice. Subsequently, samples were centrifuged at 10,000× *g* for 15 min at 4 °C, and the supernatant was collected. Nuclear and cytoplasmic protein fractions were obtained from cell pellets using the NE-PER Nuclear and Cytoplasmic Extraction Kit (78835, Thermo Fisher Scientific, Waltham, MA, USA), according to the manufacturer’s instructions. Protein concentration was determined using the Bradford assay. Equal amounts of protein (20 μg) were separated on a 4–20% SDS-PAGE gel and transferred to a PVDF membrane. Membranes were blocked with 3% bovine serum albumin (BSA) diluted in Tris-buffered saline containing 0.1% Tween-20 and then incubated overnight at 4 °C with primary antibodies against: Zona occludens 1 (ZO-1, sc-33725, Santa Cruz Biotechnology, Dallas, TX, USA), claudin 1 (37-4900, Invitrogen, Thermo Fisher Scientific, Waltham, MA, USA), claudin 3 (34-1700, Invitrogen, Thermo Fisher Scientific, Waltham, MA, USA), p-STAT3 (ab-76315, Abcam, Cambridge, UK); STAT3 (ab-68153, Abcam, Cambridge, UK); p-ERK (sc-7383, Santa Cruz Biotechnology, Dallas, TX, USA); ERK2 (sc-1647, Santa Cruz Biotechnology, Dallas, TX, USA); NF-kB (sc-8008, Santa Cruz Biotechnology, Dallas, TX, USA); GAPDH (5174S, Cell Signaling Technology, Danvers, MA, USA); Lamin B1 (PA5-19468, Invitrogen, Thermo Fisher Scientific, Waltham, MA, USA); α-Tubulin (T6199, Sigma-Aldrich, Merck, Darmstadt, Germany). After washing, membranes were incubated with the appropriate HRP-conjugated secondary antibodies. Protein bands were visualized using iBright™ CL750 Imaging System (Invitrogen by Thermo Fisher Scientific, Waltham, MA, USA). Densitometric analysis, expressed as optical density (OD), was performed using iBright™ analysis software (Thermo Fisher Scientific Connect Platform, https://apps.thermofisher.com, Thermo Fisher Scientific, Waltham, MA, USA). Original figures can be found in Supplementary Materials.

### 2.9. Confocal Immunofluorescence

Monolayers of Caco-2 cells were fixed with 4% PFA for 10 min and permeabilized with 0.1% Triton X-100 for 10 min. After three washings with PBS-T (PBS containing 0.1% Tween-20), samples were then blocked with 2% BSA in PBS-T and subsequently incubated overnight at 4 °C with the following primary antibodies diluted in 0.2% BSA in PBS-T: claudin-1 Monoclonal Antibody (2H10D10), 1:100 (#37-4900, Invitrogen, Thermo Fisher Scientific, Waltham, MA, USA); claudin-2 Polyclonal Antibody (MH44), 1:100 (#51-6100, Invitrogen, Thermo Fisher Scientific, Waltham, MA, USA); claudin-3 Polyclonal Antibody, 1:100 (#34-1700, Invitrogen, Thermo Fisher Scientific, Waltham, MA, USA); ZO-1 Antibody (R40.76), 1:70 (#sc-33725, Santa Cruz Biotechnology, Dallas, TX, USA). After washing, samples were incubated with appropriate secondary antibodies, diluted in 0.2% BSA in PBS-T: Alexa Fluor™ 488 Goat anti-Rat IgG (H + L), 1:500 (#A-11006, Invitrogen, Thermo Fisher Scientific, Waltham, MA, USA); Alexa Fluor™ 488 Goat anti-Mouse IgG (H + L), 1:750 (#A-11029, Invitrogen, Thermo Fisher Scientific, Waltham, MA, USA); Alexa Fluor™ 488 Goat anti-Rabbit IgG (H + L), 1:500 (#A-11008, Invitrogen, Thermo Fisher Scientific, Waltham, MA, USA); Alexa Fluor™ 555 Goat anti-Rat IgG (H + L), 1:300 (#A-21434, Invitrogen, Thermo Fisher Scientific, Waltham, MA, USA); Alexa Fluor™ 555 Goat anti-Mouse IgG (H + L), 1:300 (#A-21425, Invitrogen, Thermo Fisher Scientific, Waltham, MA, USA). Nuclei were counterstained with 4′,6-diamidino-2-phenylindole (DAPI, 5 µg/mL). Imaging was performed using a confocal laser-scanning microscope, Leica TCS SP8 (Leica Microsystems, Mannheim, Germany), and fluorescence quantification at the plasma membrane was carried out using ImageJ software (Version 1.54p, NIH, Bethesda, MD, USA).

### 2.10. Statistical Analysis

All experiments were performed with at least three independent biological replicates and three technical replicates. Data were analyzed using GraphPad Prism software (version 7.0; GraphPad Software Inc., San Diego, CA, USA) and are presented as mean ± standard deviation (SD). Outliers were identified and excluded using the ROUT test. Normality of data distribution was assessed with the Shapiro–Wilk test. Depending on the data distribution, statistical significance was evaluated using an unpaired *t*-test or a two-way ANOVA for parametric data, and Mann–Whitney tests for non-parametric data. For multiple comparisons, all groups were compared with each other using Tukey’s (parametric) post-hoc test. A *p*-value < 0.05 was considered statistically significant.

## 3. Results

### 3.1. Ultrastructural Remodeling of Tight Junctions, Lipid Accumulation, and ROS Production Under Simulated Mars Gravity

**Figure 1:**
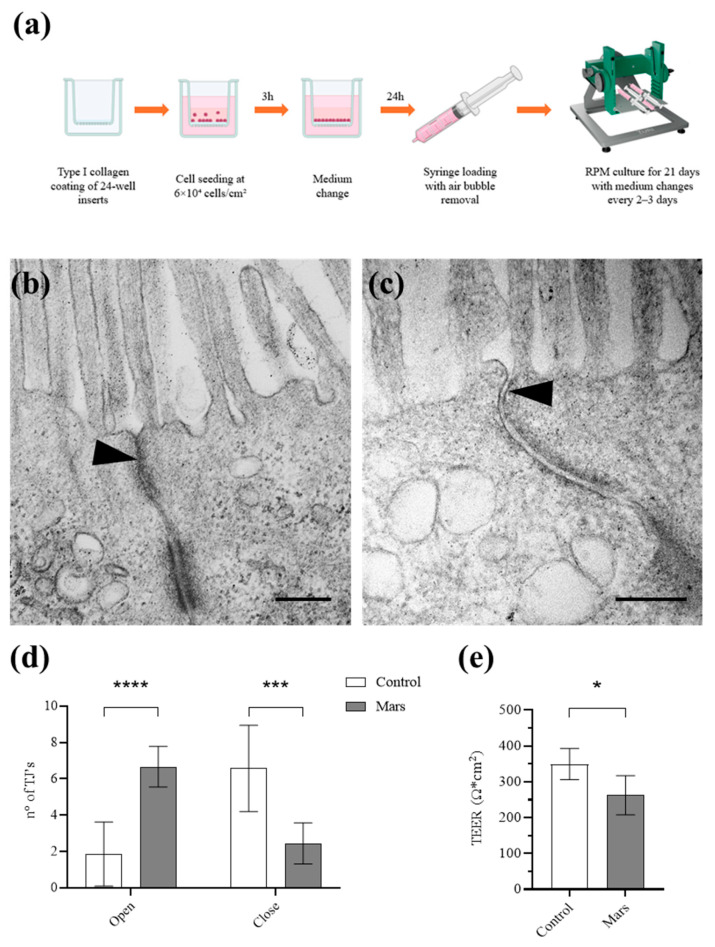
(**a**) Scheme depicting the experimental workflow followed to grow a Caco-2 cell monolayer in simulated Mars gravity. (**b**) Representative control Caco-2 cell showing a «closed» Tight Junction (TJ) (indicated by arrowhead) by Transmission Electron Microscopy (TEM); (**c**) Representative reduced gravity-cultured Caco-2 cell showing an «open» TJ (indicated by arrowhead) by TEM; (**d**) Graph illustrating the number of open and closed TJs in control cells (white) and simulated Mars gravity (gray). Each bar represents the mean values (±SD) derived from eight independent experiments. **** = *p* < 0.0001, *** = *p* < 0.001 calculated by two-way ANOVA. (**e**) Graph illustrating the mean TEER value (±SD) measured in control (white) and simulated Mars gravity (gray) monolayers. Each bar represents the mean values (±SD) derived from three independent experiments. * = *p* < 0.05 calculated by unpaired *t*-test. Scale bars are 200 nm in (**b**) and 400 nm in (**c**).

To investigate the effects of simulated Mars gravity on intestinal barrier integrity, Caco-2 cells were cultured for 15 or 21 days under simulated gravity conditions obtained using RPM, a ground-based model to simulate microgravity [22], after which cellular morphology was analyzed by transmission electron microscopy (TEM) (Figure 1a–c). In control cells maintained at 1 g, well-defined TJ were observed only after 21 days of differentiation, localized to the apical-most domain of the epithelium, and characterized by the typical ultrastructural hallmark of TJ formation, namely the fusion of adjacent plasma membranes forming the so-called “kissing points” (here called closed TJ) (Figure 1b). Based on these observations, the 21-day differentiation time point was selected for subsequent experiments. Ultrastructural analysis revealed that simulated Mars gravity alters TJ architecture. Specifically, kissing points were largely absent, resulting in predominantly open TJ and a marked widening of the paracellular space (Figure 1c). Quantitative analysis further demonstrated a significant reduction in the number of closed TJs under microgravity conditions compared with 1 g controls (Figure 1d). TEER analysis, used to measure TJ integrity, indicated a reduction in transepithelial electrical resistance in reduced gravity-exposed cells (Figure 1e).

**Figure 2:**
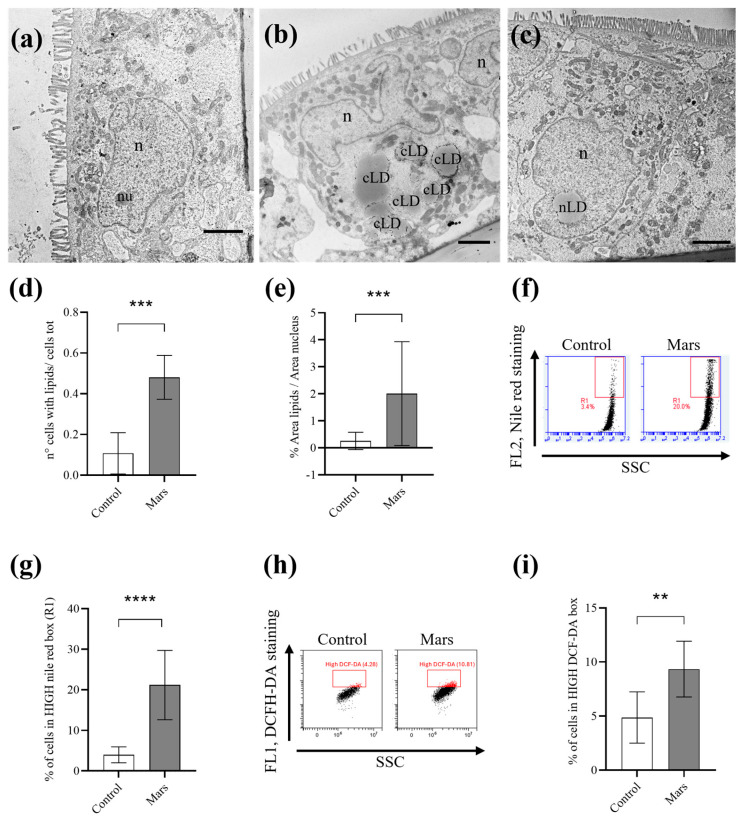
Effect of simulated Mars gravity on cytoplasmic (cLD) and nuclear (nLD) lipid droplet content in Caco-2 cells. (**a**) Representative control Caco-2 cells without cLDs and nLDs. (**b**) Representative simulated Mars gravity-cultured Caco-2 cells showing multiple cLDs. (**c**) Representative simulated Mars gravity-cultured Caco-2 cells showing an nLD. (**d**) Graph illustrating the number of cells with nLDs on total cells counted in control (white) and simulated Mars gravity (grey) samples. Each bar represents the mean value (±SD) derived from six independent experiments. *** = *p* < 0.001 calculated by unpaired *t*-test. (**e**) Graph illustrating the percentage of the nucleus area occupied by nLDs in control cells (white) and simulated Mars gravity (grey). Each bar represents the mean value (±SD) derived from six independent experiments. *** = *p* < 0.001 calculated by the Mann–Whitney test. (**f**) Representative cytometry plots displaying Nile red-stained control and simulated Mars gravity-cultured Caco-2 cells. (**g**) Graph illustrating the percentage of cells exhibiting high Nile red content (within the HIGH Nile red box). Each bar represents the mean values (±SD) evaluated in four independent experiments. **** = *p* < 0.0001 calculated by unpaired *t*-test. (**h**) Representative cytometry plots displaying DCFH-DA-stained control and simulated Mars gravity-cultured Caco-2 cells. (**i**) Graph illustrating the percentage of cells within the HIGH DCFH-DA box. Each bar represents the mean values (±SD) evaluated in four independent experiments. ** = *p* < 0.01 calculated by unpaired *t*-test. Scale bar is 5 μm in (**a**–**c**). n, nucleus; nu, nucleolus.

TEM analysis also revealed a significant accumulation of lipid droplets (LD) in both the cytoplasm and the nucleus of Caco-2 cells under simulated Mars gravity conditions (Figure 2a–e). This increase in intracellular lipid content was confirmed by Nile Red staining, which showed a marked enhancement of lipids in microgravity-exposed cells compared with 1 g controls (Figure 2f,g).

As microgravity is known to induce oxidative stress in cells, we quantified ROS production in Caco-2 cells exposed to simulated Mars gravity for 21 days by DCFH-DA assay using the lipophilic non-fluorescent dye that becomes fluorescent in the presence of ROS. As shown in Figure 2h,i, the percentage of cells with a high fluorescent signal is increased in cells under simulated Mars gravity with respect to the control, indicating an increase in ROS production.

### 3.2. Selective Modulation of Tight Junction Component Expression Under Simulated Mars Gravity

**Figure 3:**
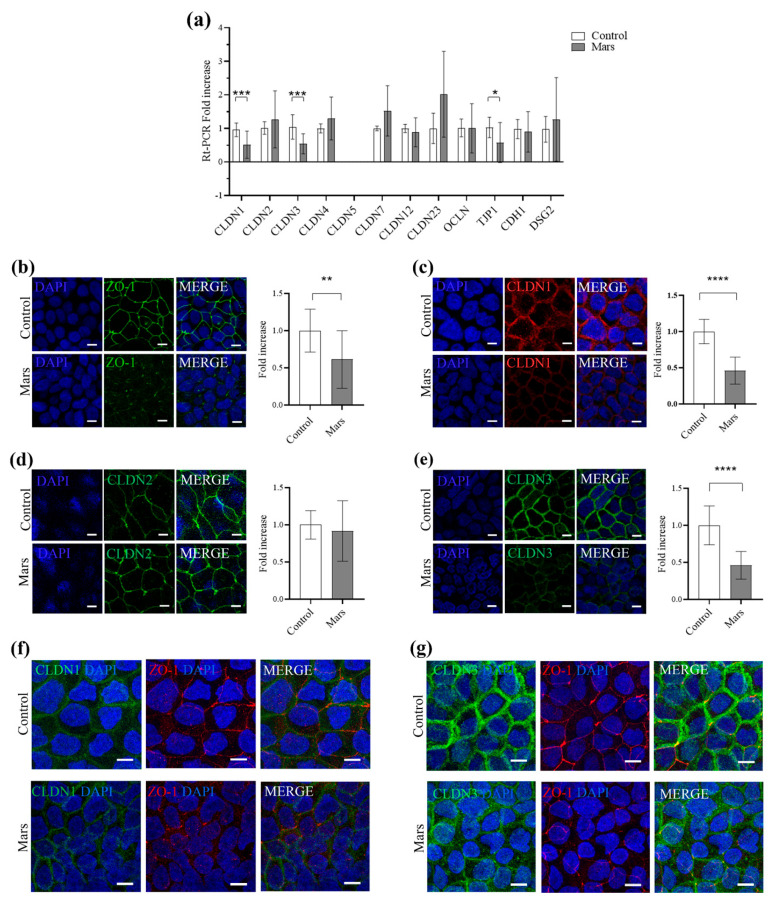
(**a**) Graph illustrating the Real-time PCR analysis of the gene expression of *claudin-1* (*CLDN1*), *claudin-2* (*CLDN2*), *claudin-3* (*CLDN3*), *claudin-4* (*CLDN4*), *claudin-5* (*CLDN5*), *claudin-7* (*CLDN7*), *claudin-12* (*CLDN12*), *claudin-23* (*CLDN23*), *occludin* (*OCLN*), *tight junction protein 1* (*TJP1*, encoding for ZO-1), *E-cadherin* (*CDH1*), and *desmoglein-2* (*DSG2*) in control cells (white) and simulated Mars gravity cells (grey). Each bar represents the mean value (±SD) derived from five independent experiments. * = *p* < 0.05, *** = *p* < 0.001 calculated by the Mann–Whitney test. (**b**) Representative confocal images of control and simulated Mars gravity monolayers stained for ZO-1 (green) and DAPI (blue). Graph illustrating the fold increase in fluorescence in control cells (white) and simulated Mars gravity cells (grey). Each bar represents the mean value (±SD) derived from four independent experiments. ** = *p* < 0.01 calculated by unpaired *t*-test. (**c**) Representative confocal image of control and simulated Mars gravity monolayer stained for CLDN1 (red) and DAPI (blue). Graph illustrating the fold increase in fluorescence in control cells (white) and simulated Mars gravity cells (grey). Each bar represents the mean value (±SD) derived from five independent experiments. **** = *p* < 0.0001 calculated by unpaired *t*-test. (**d**) Representative confocal image of control and simulated Mars gravity monolayer stained for CLDN2 (green) and DAPI (blue). Graph illustrating the fold increase in fluorescence in control cells (white) and simulated Mars gravity cells (grey). Each bar represents the mean value (±SD) derived from three independent experiments. *p*-value calculated by Unpaired *t*-test. (**e**) Representative confocal image of control and simulated Mars gravity monolayer stained for CLDN3 (green) and DAPI (blue). Graph illustrating the fold increase in fluorescence in control cells (white) and simulated Mars gravity cells (grey). Each bar represents the mean value (±SD) derived from seven independent experiments. **** = *p* < 0.0001 calculated by unpaired *t*-test. (**f**,**g**) Representative confocal images of control and simulated Mars gravity monolayers stained for CLDN1 or CLDN3 (green)/ZO-1 (red) double immunofluorescence with DAPI (blue). Scale bar is 10 μm in (**b**–**g**).

Since simulated Mars gravity alters TJ ultrastructure in differentiated Caco-2 cells, we investigated whether reduced gravity affects the expression of TJ components, with particular focus on members of the claudin (CLDN) family. We analyzed the expression of representative claudins, including barrier-forming claudins (e.g., CLDN1 and CLDN3) and pore-forming claudins that function as size- and charge-selective ion/water channels (e.g., CLDN2) [23,24]. Real-time PCR analysis demonstrated that microgravity induced a significant downregulation of *CLDN1* and *CLDN3*, whereas the expression of *CLDN2*, *CLDN4*, *CLDN7*, *CLDN12*, and *CLDN23* was not affected (Figure 3a). As reported in the literature, *CLDN5* is not expressed in Caco-2 cells [25].

Among other TJ components, *occludin* (*OCLN*) expression was not altered, whereas *TJP1* expression, encoding ZO-1, was significantly reduced under microgravity conditions. No significant changes were detected in the expression of *E-cadherin* (*CDH1*), a canonical marker of adherens junctions, or *desmoglein-2* (*DSG2*), a key component and marker of desmosomal junctions (Figure 3a).

**Figure 4:**
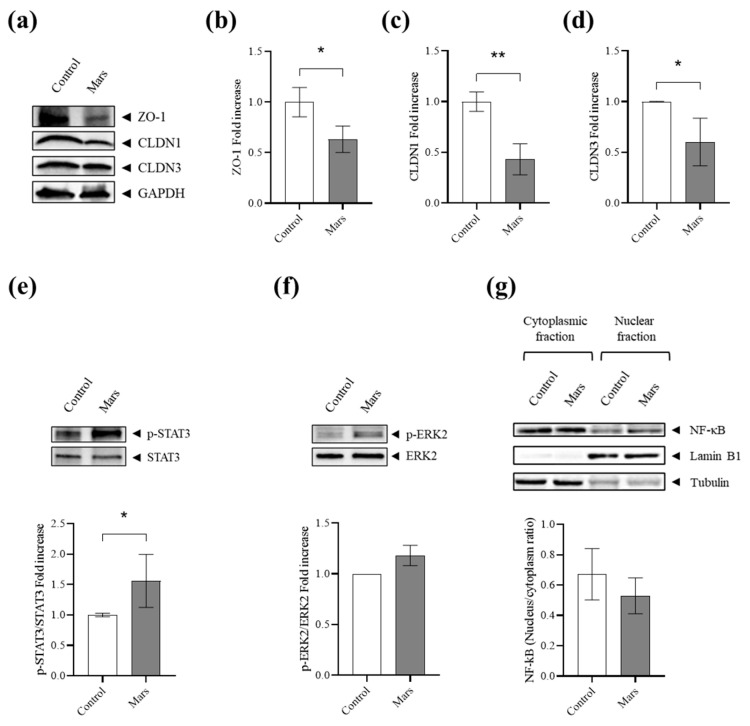
(**a**) Representative Western blot of Zona occludens 1 (ZO-1), claudin-1 (CLDN1), and claudin-3 (CLDN3) in control or Mars gravity-cultured Caco-2 cells. (**b**) Graph illustrating the fold increase of densitometric analysis of ZO-1 expression levels in control cells (white) and simulated Mars gravity cells (grey). Each bar represents the mean value (±SD) derived from three independent experiments. * = *p* < 0.05 calculated by unpaired *t*-test. (**c**) Graph illustrating the fold increase of densitometric analysis of CLNDN1 expression levels in control cells (white) and simulated Mars gravity cells (grey). Each bar represents the mean value (±SD) derived from three independent experiments. ** = *p* < 0.01 calculated by unpaired *t*-test. (**d**) Graph illustrating the fold increase of densitometric analysis of CLDN3 expression levels in control cells (white) and simulated Mars gravity cells (grey). Each bar represents the mean value (±SD) derived from three independent experiments. * = *p* < 0.05 calculated by unpaired *t*-test. (**e**) Representative blot and graph illustrating the fold increase of densitometric analysis of pSTAT3/STAT3 expression levels in control cells (white) and simulated Mars gravity cells (grey). Each bar represents the mean value (±SD) derived from three independent experiments. * = *p* < 0.05 calculated by unpaired *t*-test. (**f**) Representative blot and graph illustrating the fold increase of densitometric analysis of pERK2/ERK2 expression levels in control cells (white) and simulated Mars gravity cells (grey). Each bar represents the mean value (±SD) derived from three independent experiments. *p*-value calculated by unpaired *t*-test. (**g**) Representative blot and corresponding graph illustrating the NF-κB expression level ratio between nuclear and cytoplasmic fractions in control cells (white) and simulated Mars gravity cells (grey). Each bar represents the mean value (±SD) derived from three independent experiments. *p*-value calculated by Unpaired *t*-test.

Immunofluorescence analysis corroborated the decreased expression of these TJ components, without evidence of significant protein mislocalization in simulated Mars gravity, as CLDN1, CLDN3, and ZO-1 remained predominantly localized at the plasma membrane, comparable to their distribution in 1 g control cells (Figure 3b–e). In fully differentiated cells in microgravity, ZO-1 still colocalizes with CLDN1 and CLDN3 at the plasma membrane (Figure 3f,g). To validate the downregulation of CLDN1, CLDN3, and ZO-1 at the protein level, we performed a Western blot assay, which revealed a significant reduction in the levels of these proteins in Caco-2 cells after 21 days of simulated Mars gravity (Figure 4a,d).

### 3.3. Selective Activation of the STAT3 Pathway Under Simulated Mars Gravity Conditions

To investigate how oxidative stress may contribute to the downregulation of CLDN1, CLDN3, and ZO-1, we analyzed the expression and activation status of key components of ROS-sensitive signaling pathways, including STAT3, NF-Κb, and ERK2 [26,27].

Western blot analysis showed that reduced gravity induces a significant increase in phosphorylated STAT3 (p-STAT3) relative to its total form, indicating activation of the STAT3 pathway. In contrast, no significant changes were observed in the activation status of ERK2 or NF-κB (Figure 4e–g).

### 3.4. Impaired De Novo Tight Junction Assembly Under Simulated Mars Gravity

**Figure 5:**
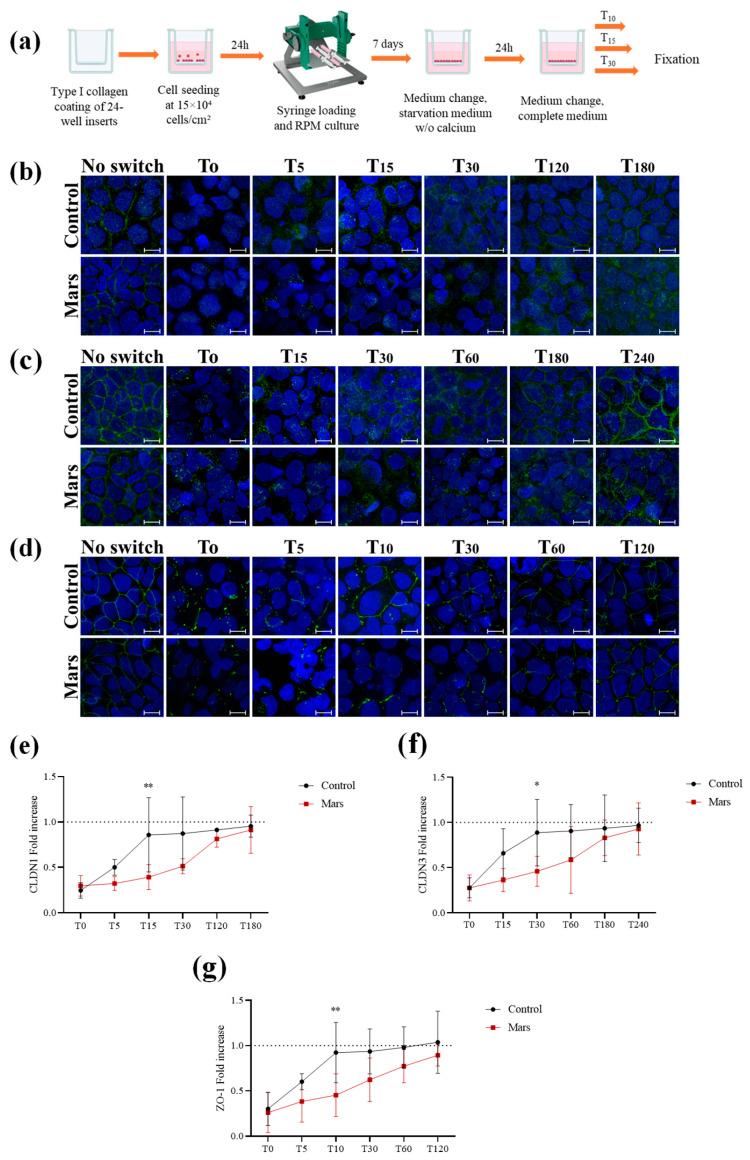
(**a**) Scheme depicting the experimental workflow followed to perform Calcium Switch Assay on a Caco-2 cell monolayer in simulated Mars gravity. (**b**) Representative confocal image of control and simulated Mars gravity monolayer stained for CLDN1 (green) and DAPI (blue) after Calcium Switch Assay. (**c**) Representative confocal image of control and simulated Mars gravity monolayer stained for CLDN3 (green) and DAPI (blue) after Calcium Switch Assay. (**d**) Representative confocal image of control and simulated Mars gravity monolayer stained for ZO-1 (green) and DAPI (blue) after Calcium Switch Assay. (**e**) Graph illustrating the fold increase of CLDN1 fluorescence in control cells (black) and simulated Mars gravity cells (red) with respect to T0, at different time points after Calcium Switch Assay. Each point represents the mean value (±SD) derived from three independent experiments. ** = *p* < 0.01 calculated by two-way ANOVA. (**f**) Graph illustrating the fold increase of CLDN3 fluorescence in control cells (black) and simulated Mars gravity cells (red) with respect to T0, at different time points after Calcium Switch Assay. Each bar represents the mean value (±SD) derived from three independent experiments. * = *p* < 0.05, calculated by two-way ANOVA. (**g**) Graph illustrating the fold increase of ZO-1 fluorescence in control cells (black) and simulated Mars gravity cells (red) with respect to T0, at different time points after Calcium Switch Assay. Each bar represents the mean value (±SD) derived from three independent experiments. ** = *p* < 0.01 calculated by two-way ANOVA. Scale bar is 10 μm in (**b**–**d**).

To investigate whether reduced gravity affects de novo TJ protein assembly, we performed a calcium switch assay in Caco-2 cells exposed to simulated Mars gravity for one week (Figure 5a). After only one week of microgravity exposure, Caco-2 cells already showed reduced CLDN1, CLDN3, and ZO-1 expression compared with control cells. Following TJ disassembly (T_0_), the expression of these proteins was further reduced compared with controls, and their relocalization at the plasma membrane during TJ reassembly was delayed in comparison to 1 g cells (Figure 5b–g). CLDN1, CLDN3, and ZO-1 generally begin to relocalize at the plasma membrane of some cells, forming strand-like structures after 15, 30, and 10 min, respectively, whereas incomplete reassembly was still detected in cells exposed to reduced gravity at these time points, with a predominantly cytoplasmic expression of TJ proteins. Although the exact timing varied between experiments, most cells exposed to Mars gravity showed a ZO-1 spatial expression pattern similar to controls after 60 min, a CLDN3 expression pattern after 240 min, and a CLDN1 expression pattern after 180 min following the switch.

## 4. Discussion

A growing body of evidence indicates that long-duration spaceflight conditions challenge epithelial barrier homeostasis through combined mechanical unloading and oxidative stress [28]. Indeed, microgravity exposure significantly increased intracellular ROS [7,29], which are known to destabilize TJ both by directly modifying junctional proteins and by activating redox-sensitive signaling pathways that regulate cytoskeletal dynamics and actomyosin contractility at 1 g [30,31].

In the present study, simulated Mars gravity analog conditions were generated using an RPM, which does not generate a constant reduced gravity vector (e.g., 0.38 g) but instead creates a time-averaged reduced-gravity condition through continuous random reorientation of the samples with respect to the Earth’s gravity vector [32]. Therefore, this approach does not reproduce a true constant partial gravity environment, such as Mars gravity. Nevertheless, RPM is commonly used and well-established as a ground-based platform to simulate microgravity conditions and to investigate reduced-gravity-related biological responses [33,34].

We found that RPM-simulated Mars gravity induces ROS production and morphological defects in fully differentiated Caco-2 cells, a widely accepted in vitro model of the intestinal epithelial barrier that reproduces key morphological and functional features of mature absorptive enterocytes, including the development of a brush border comparable to that of the small intestine [32].

Simulated Mars gravity-exposed Caco-2 cells showed a widening of the paracellular space due to predominantly open TJs, losing the typical “kissing points”. Together with TEER measurements, these morphological TJ alterations demonstrate a functional impairment of epithelial barrier integrity under altered gravity conditions and are in agreement with previous reports showing defects in TJ morphology and function under different simulated microgravity conditions [18,19].

Moreover, cells exposed to simulated Mars gravity showed a significant accumulation of both cytoplasmic and nuclear lipid droplets. This increase in lipid droplets is consistent with the extensive remodeling of lipid metabolism previously reported in Caco-2 cells exposed to different simulated reduced gravity, including alterations in phospholipids and sphingolipids indicative of enhanced lipid synthesis and redistribution [33]. Although nuclear lipid droplets (nLDs) are known to increase in abundance in differentiated Caco-2 cells even in the absence of external lipid supplementation at 1 g [34], reduced gravity potentiates this phenomenon, suggesting that nLD expansion may represent an adaptive response to changes in lipid homeostasis. Further studies will be necessary to elucidate the functional significance and regulatory mechanisms underlying this response.

Simulated Mars gravity appears to promote a selective reorganization of claudin expression, rather than causing a broad downregulation of TJ components or spatial defects in junctional protein localization. In this context, the barrier-forming claudins CLDN1 and CLDN3 were significantly reduced at both the mRNA and protein levels, whereas the pore-forming or context-dependent claudins CLDN2, CLDN4, CLDN7, CLDN12, and CLDN23 remained unchanged.

This selective modulation is likely to be functionally relevant, as CLDN1 and CLDN3 are key determinants of epithelial barrier integrity, contributing to junctional tightening and restriction of paracellular macromolecular flux [14], and their downregulation is consistent with the ultrastructural widening of TJs in the intestine. Notably, in vivo evidence under reduced gravity conditions is currently limited and mainly concerns CLDN1, whereas, to the best of our knowledge, in vivo data specifically addressing CLDN3 expression under real or simulated microgravity conditions are still lacking. A significant reduction in CLDN1 expression has been reported in the intestine of astronauts during spaceflight [35], and similar alterations have been described in hindlimb-unloaded rats, where decreased CLDN1 levels are associated with increased intestinal permeability and barrier dysfunction [8].

Notably, CLDN2, a pore-forming claudin frequently upregulated in inflammatory bowel disease and associated with a “leaky” epithelial phenotype, was not induced under simulated Mars gravity conditions. This observation suggests that reduced gravity-associated barrier impairment may differ mechanistically from classical inflammatory pathologies, which are typically characterized by CLDN2 induction and pore formation rather than selective loss of structural, barrier-forming claudins [36].

Among other TJ components, ZO-1 expression was significantly reduced, whereas occludin levels remained unchanged. ZO-1 is a critical scaffolding protein that physically links transmembrane TJ proteins, including claudins and occludin, to the actin cytoskeleton and plays a central role in TJ assembly, maturation, and barrier integrity [37]. Indeed, ZO-1 proteins self-organize into membrane-associated compartments through phase separation and are required for the assembly of functional TJs [38]. ZO-1 downregulation in Mars gravity is therefore consistent with impaired TJ stability and incomplete junctional maturation under microgravity conditions that might reflect compromised mechanotransduction at cell–cell junctions, given that microgravity is known to profoundly affect cytoskeletal organization and mechanosensitive signaling pathways [39].

Activation of STAT3, in the absence of ERK signaling or NF-κB activation, is consistent with a stress-adaptive rather than a canonical inflammatory response to reduced gravitational loading. Indeed, STAT3 is a well-established redox-sensitive transcription factor whose activation can be triggered by increased intracellular ROS independently of pro-inflammatory signaling [40]. Although STAT3 is generally considered protective in intestinal epithelial cells [41,42], its effects appear to be context-dependent, as in endothelial and epithelial systems, ROS-driven STAT3 activation has been shown to modulate junctional stability by regulating the expression of barrier-forming proteins [43].

The selective downregulation of CLDN1 and CLDN3, together with preserved expression of CLDN2 and other pore-forming claudins, may be compatible with a STAT3-associated remodeling program that weakens junctional tightening without eliciting a classical inflammatory “leaky” phenotype; however, our data do not allow us to establish a causal relationship, and alternative regulatory mechanisms cannot be excluded. In this context, STAT3 activation may reflect a compensatory mechanism to maintain epithelial barrier integrity, and oxidative stress–induced damage may override its potential protective role, resulting in the observed loss of barrier-forming tight junction proteins.

Notably, NF-κB signaling is a key mediator of inflammatory responses and has been implicated in CLDN2 induction and barrier disruption [44]. Therefore, the lack of NF-κB activation in our model supports the absence of a canonical inflammatory response, although it does not fully account for the regulation of CLDN2, which is known to be controlled by multiple pathways [45].

Beyond transcriptional control, STAT3 has been implicated in the regulation of cytoskeletal organization and actomyosin dynamics, processes essential for both TJ maintenance and de novo assembly [46]. Accordingly, simulated Mars gravity not only destabilized mature TJs but also impaired junctional reassembly, as evidenced by reduced expression and delayed membrane relocalization of CLDN1, CLDN3, and ZO-1 in calcium switch assays. These defects in TJ biogenesis are particularly relevant for the intestinal epithelium, which undergoes continuous cell turnover and depends on rapid TJ reformation to preserve barrier integrity [11]. While further studies using STAT3 inhibition will be required to establish direct causality, our findings suggest a model in which simulated Mars gravity induces oxidative stress–driven STAT3 activation, selective claudin loss, and defective TJ remodeling.

## 5. Conclusions

In conclusion, to our knowledge, these data provide the first evidence in an intestinal epithelial model that simulated Mars gravity promotes oxidative stress-associated TJ remodeling, supporting the preservation of TJ integrity as a potential countermeasure strategy. Although the in vitro nature of this model requires caution when extrapolating to in vivo conditions—where additional systemic and microenvironmental factors may influence epithelial responses—our observations suggest that simulated Mars gravity can influence intestinal epithelial barrier integrity through stress-adaptive mechanisms distinct from classical inflammatory barrier disruption. These observed changes may create conditions that favor increased permeability and inflammatory priming. In this context, our findings provide a mechanistic framework that may help to interpret intestinal dysfunction and immune alterations reported in spaceflight models and astronauts, highlighting the preservation of TJ homeostasis as a critical objective for maintaining gut integrity during long-duration missions, including future human exploration of Mars.

## Data Availability

The original contributions presented in this study are included in the article/Supplementary Material. Further inquiries can be directed to the corresponding authors.

## Supplementary Materials

The following supporting information can be downloaded at: https://www.mdpi.com/article/10.3390/biom16050739/s1, Table S1: Sequences of primers used for the real-time PCR.
