# Supplementary material for: Simulated Mars Gravity Impairs Intestinal Epithelial Barrier Integrity via Selective Modulation of Tight Junction Components
Source: Biomolecules. 2026 May 18;16(5):739. doi: 10.3390/biom16050739 (PMC13204386; doi:10.3390/biom16050739)
Supplement: Supplementary file 1 [file biomolecules-16-00739-s001.zip › biomolecules-4197591-Table S1.pdf]

Table S1. Sequences of primers used for the real-time PCR.

Name	Sequence
Claudin-1 (CLDN1)	FW 5’-GACAACATCGTGACCGCCCA-3’<br>RV 5’-AACCACCATCAAGGCACGGG-3’
Claudin-2 (CLDN2)	FW 5’-AGTTCTTATGTCGGTGCCAGC-3’<br>RV 5’-TGCTATAGATGTCACACTGGGT-3’
Claudin-3 (CLDN3)	FW 5’-TTCTCGCCGCCCTGCTCAC-3’<br>RV 5’-CCGCCCAGCCCACGTACAG-3’
Claudin-4 (CLDN4)	FW 5’-GCTTTATCTCCTGACTCACGG-3’<br>RV 5’-TGAGGACCTGGAAGGCTGAG-3’
Claudin-5 (CLDN5)	FW 5’-ACCTGGAAGGGGCTGTGGAT-3’<br>RV 5’-AACGAACGCCAGCAGCACGG-3’
Claudin-7 (CLDN7)	FW 5’-GCTCCTATGCGGGTGACAAC-3’<br>RV 5’-CCAGGGAGACCACCATTAGG-3’
Claudin-12 (CLDN12)	FW 5’-GATGTCCACGCAGCCACAGT-3’<br>RV 5’-TTTCTCCAGTTGGGAAGCAGA-3’
Claudin-23 (CLDN23)	FW 5’-CAGTGGACGTGGAGTTGTACC-3’<br>RV 5’-CAGCGAGGTGACCATGAGTG-3’
Occludin (OCLN)	FW 5’-GTTCGACCAATGCTCTCTCAG-3’<br>RV 5’-CACACAGGCAAAGATGGCAAT-3’
Zonula occludens-1 (TJP1)	FW 5’-CAAGAGCACAGCAATGGAGGA-3’<br>RV 5’-TTGACGTTTCCCCACTCTGAA-3’
E-cadherin (CDH1)	FW 5’-CGGACGATGATGTGAACACC-3’<br>RV 5’-TTGCTGTTGTGCTTAACCCC-3’
Desmoglein-2 (DSG2)	FW 5’-CAACAGATGCAGATGAGCCC-3’<br>RV 5’-AGTGTAGCTGCTGTGTTCCT-3’
Glyceraldehyde-3-phosphate dehydrogenase (GAPDH)	FW 5’-AAGGTGAAGGTCGGAGTCAAC-3’<br>RV 5’-GTTCTCAGCCTTGACGGTGC-3’
Hypoxanthine-guanine phosphoribosyltransferase (HPRT)	FW 5’-CCTGGCGTCGTGATTAGTG-3’<br>RV 5’-GCCTCCCATCTCCTTCATC-3’
