# Supplementary material for: Simulated Mars Gravity Impairs Intestinal Epithelial Barrier Integrity via Selective Modulation of Tight Junction Components
Source: Biomolecules. 2026 May 18;16(5):739. doi: 10.3390/biom16050739 (PMC13204386; doi:10.3390/biom16050739)

Full lenght Western Blots

p-STAT3

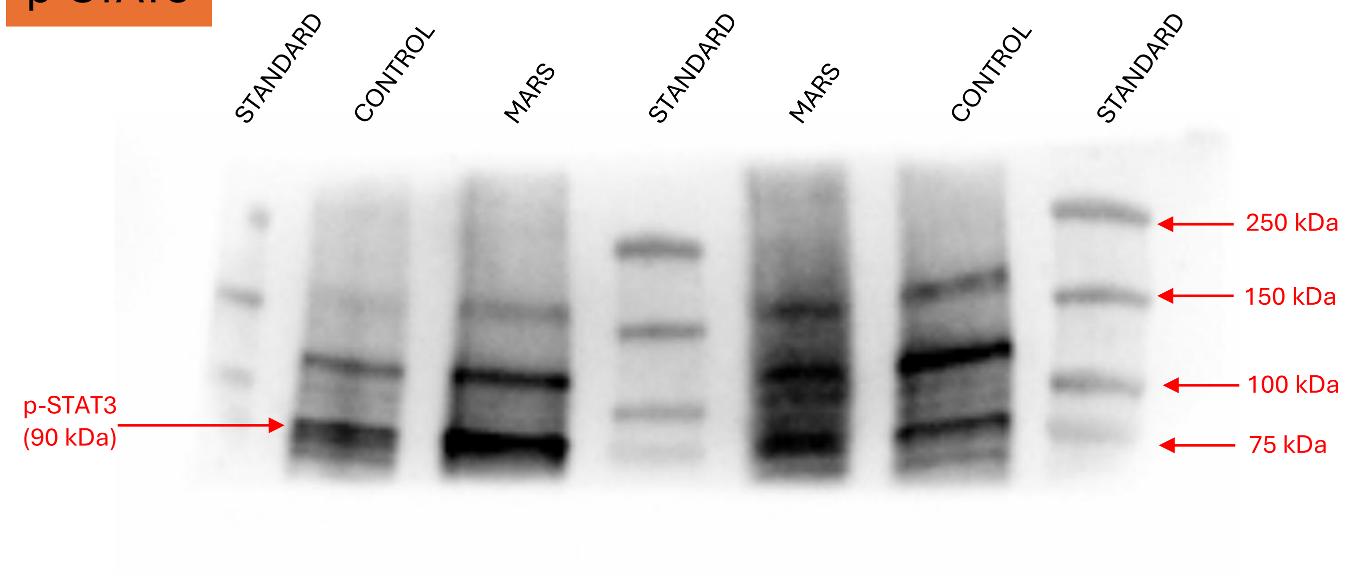

STAT3

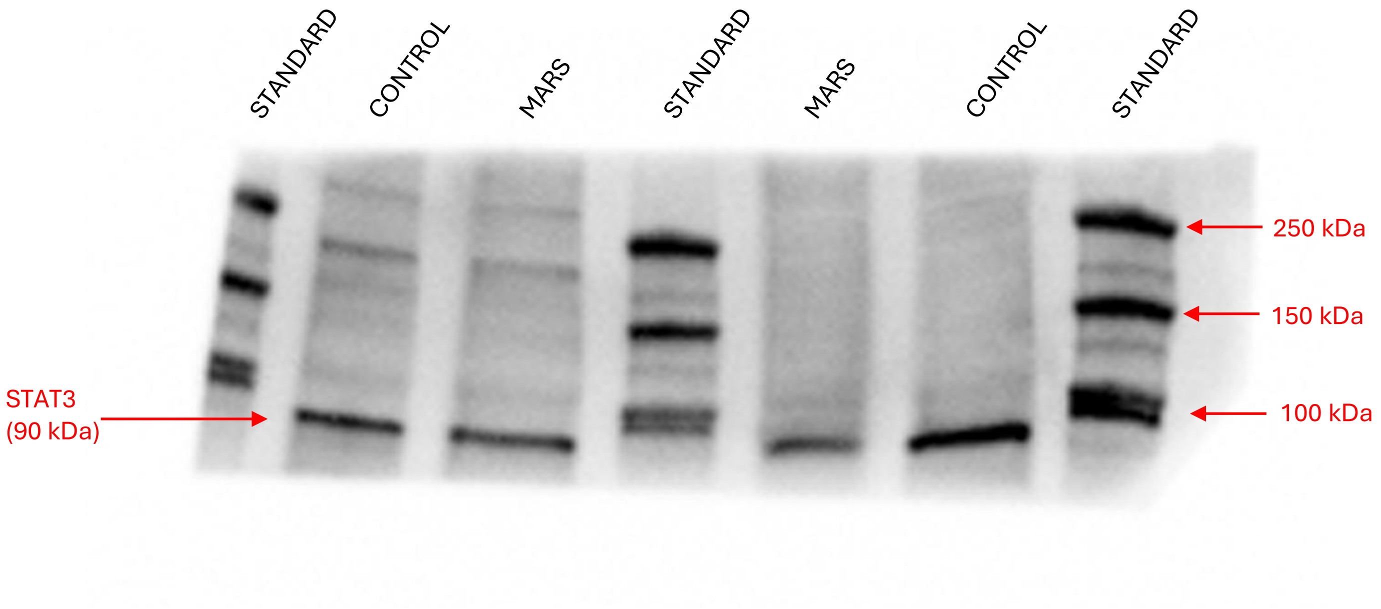

Full lenght Western Blots

p-ERK

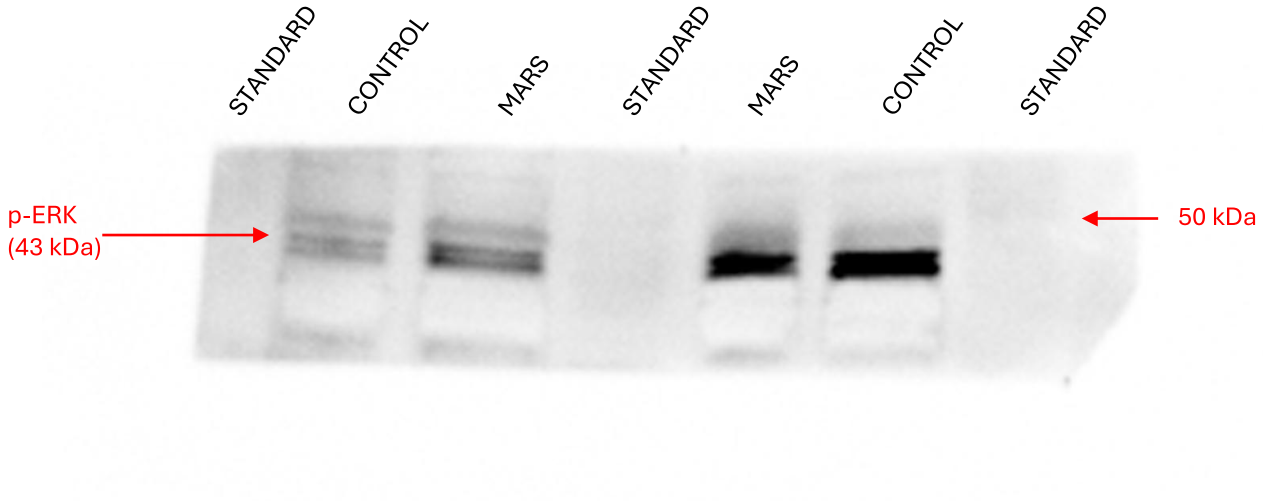

ERK2

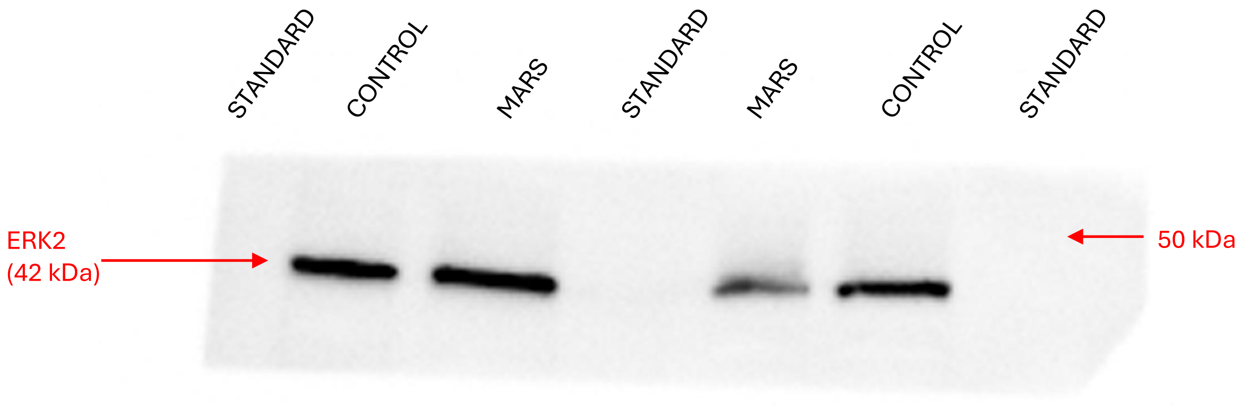

Full lenght Western Blots

ZO-1

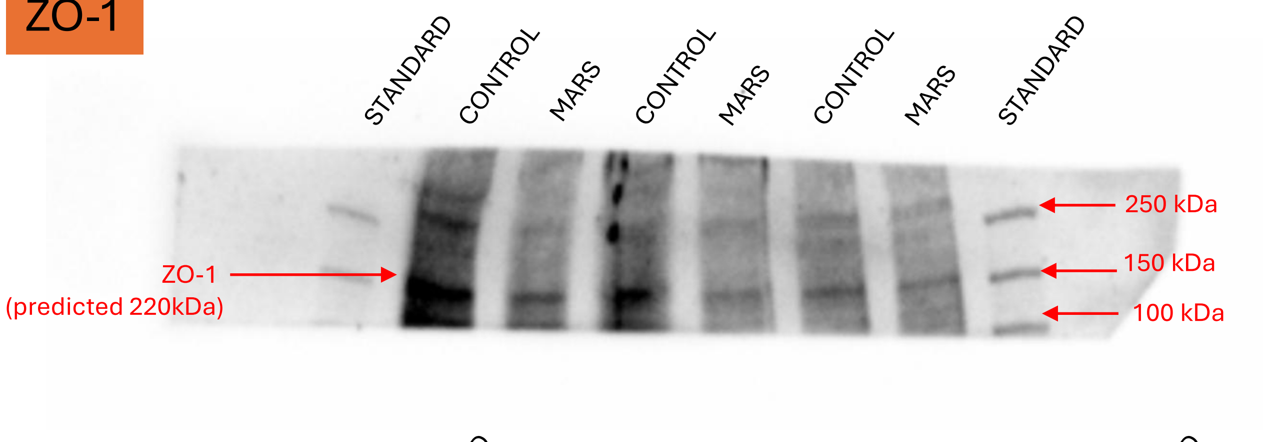

CLDN3

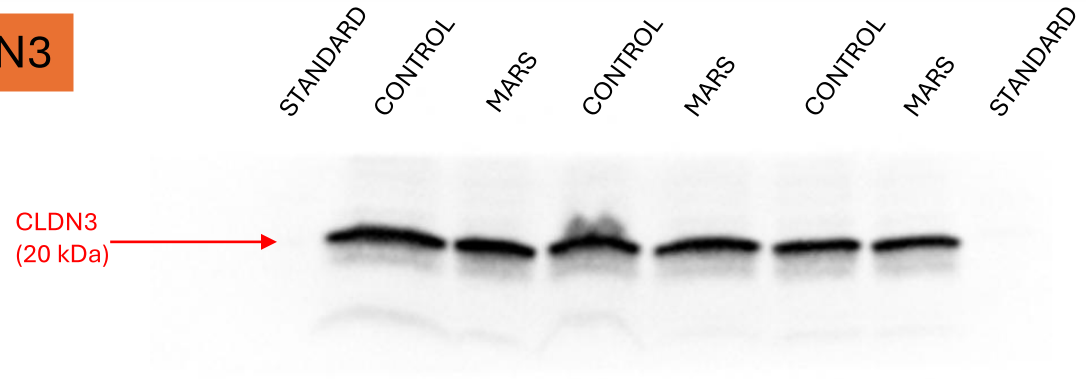

CLDN1

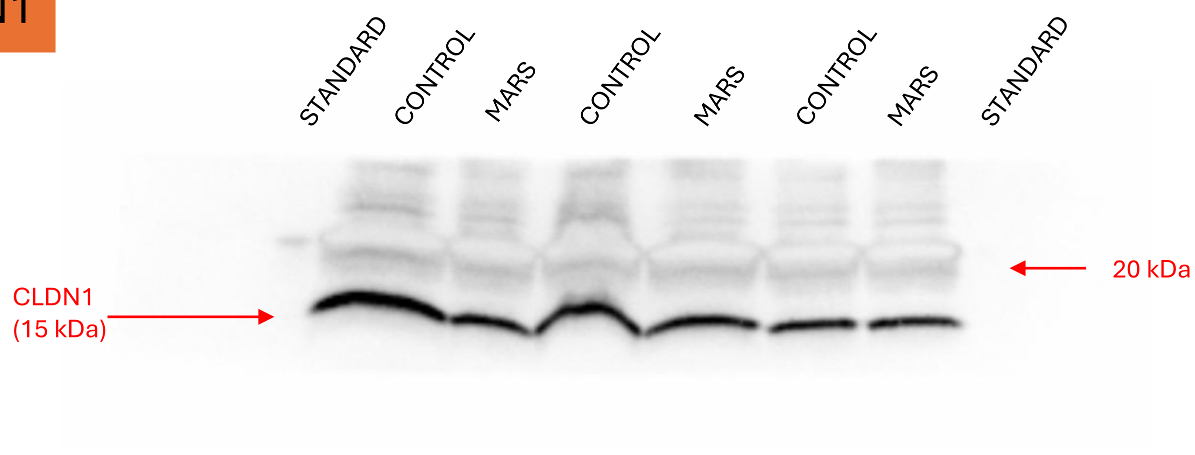

GAPDH

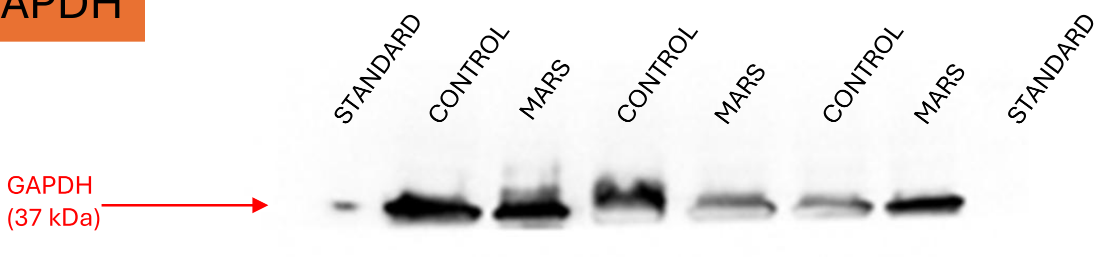

Full lenght Western Blots

GAPDH

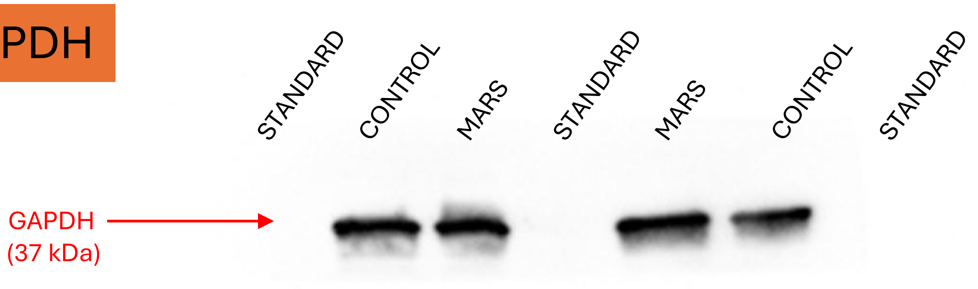

NF-κB

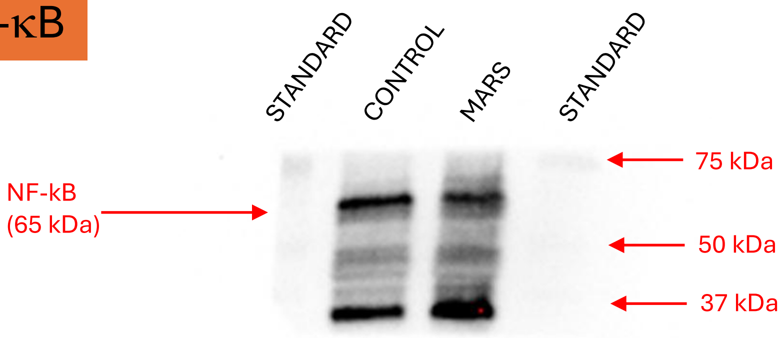

Supplement: Supplementary file 1 [file biomolecules-16-00739-s001.zip › biomolecules-4197591-WB-original.pdf]
